# Missense and loss‐of‐function variants at GWAS loci in familial Alzheimer's disease

**DOI:** 10.1002/alz.14221

**Published:** 2024-09-05

**Authors:** Tamil Iniyan Gunasekaran, Dolly Reyes‐Dumeyer, Kelley M. Faber, Alison Goate, Brad Boeve, Carlos Cruchaga, Margaret Pericak‐Vance, Jonathan L. Haines, Roger Rosenberg, Debby Tsuang, Diones Rivera Mejia, Martin Medrano, Rafael A. Lantigua, Robert A. Sweet, David A. Bennett, Robert S. Wilson, Camille Alba, Clifton Dalgard, Tatiana Foroud, Badri N. Vardarajan, Richard Mayeux

**Affiliations:** ^1^ Department of Neurology Taub Institute for Research on Alzheimer's Disease and the Aging Brain and the Gertrude H. Sergievsky Center Columbia University New York New York USA; ^2^ Department of Medical and Molecular Genetics National Centralized Repository for Alzheimer's Disease and Related Dementias (NCRAD), 410 W. 10th St., HS 4000. Indiana University School of Medicine Indianapolis Indiana USA; ^3^ Department of Genetics & Genomic Sciences Ronald M. Loeb Center for Alzheimer's disease Icahn School of Medicine at Mount Sinai Icahn Bldg., One Gustave L. Levy Place New York New York USA; ^4^ Department of Neurology, Mayo Clinic Rochester Minnesota USA; ^5^ Department of Psychiatry Washington University in St. Louis, Rand Johnson Building, 600 S Euclid Ave., Wohl Hospital Building St. Louis Missouri USA; ^6^ John P Hussman Institute for Human Genomics Dr. John T Macdonald Foundation Department of Human Genetics University of Miami Miller School of Medicine Miami Florida USA; ^7^ Department of Population & Quantitative Health Sciences and Cleveland Institute for Computational Biology. Case Western Reserve University Cleveland Ohio USA; ^8^ Department of Neurology University of Texas Southwestern Medical Center Dallas Texas USA; ^9^ Department of Psychiatry and Behavioral Sciences University of Washington, GRECC VA Puget Sound, 1660 South Columbian Way Seattle Washington USA; ^10^ Los Centros de Diagnóstico y Medicina Avanzada y de Conferencias Médicas y Telemedicina CEDIMAT, Arturo Logroño Plaza de la Salud, Dr. Juan Manuel Taveras Rodríguez, C. Pepillo Salcedo esq Santo Domingo Dominican Republic; ^11^ Universidad Pedro Henríquez Urena, Av. John F. Kennedy Km. 7‐1/2 Santo Domingo 1423 Santo Domingo Dominican Republic; ^12^ Pontíficia Universidad Católica Madre y Maestra (PUCMM), Autopista Duarte Km 1 1/2 Santiago de los Caballeros Dominican Republic; ^13^ Department of Medicine Vagelos College of Physicians and Surgeons Columbia University, and the New York Presbyterian Hospital New York New York USA; ^14^ Departments of Psychiatry and Neurology University of Pittsburgh Pittsburgh Pennsylvania USA; ^15^ Rush Alzheimer's Disease Center Rush University Medical Center, 1750, West Harrison St Chicago Illinois USA; ^16^ Department of Anatomy Physiology and Genetics Uniformed Services University of the Health Sciences Bethesda Maryland USA

**Keywords:** familial Alzheimer's disease, gene loci, genetic segregation, genome wide association studies, rare variants

## Abstract

**BACKGROUND:**

Few rare variants have been identified in genetic loci from genome‐wide association studies (GWAS) of Alzheimer's disease (AD), limiting understanding of mechanisms, risk assessment, and genetic counseling.

**METHODS:**

Using genome sequencing data from 197 families in the National Institute on Aging Alzheimer's Disease Family Based Study and 214 Caribbean Hispanic families, we searched for rare coding variants within known GWAS loci from the largest published study.

**RESULTS:**

Eighty‐six rare missense or loss‐of‐function (LoF) variants completely segregated in 17.5% of families, but in 91 (22.1%) families Apolipoprotein E (*APOE)‐*𝜀4 was the only variant segregating. However, in 60.3% of families, *APOE* 𝜀4, missense, and LoF variants were not found within the GWAS loci.

**DISCUSSION:**

Although *APOE* 𝜀4and several rare variants were found to segregate in both family datasets, many families had no variant accounting for their disease. This suggests that familial AD may be the result of unidentified rare variants.

**Highlights:**

- Rare coding variants from GWAS loci segregate in familial Alzheimer's disease.
- Missense or loss of function variants were found segregating in nearly 7% of families.
- APOE‐𝜀4 was the only segregating variant in 29.7% in familial Alzheimer's disease.
- In Hispanic and non‐Hispanic families, different variants were found in segregating genes.
- No coding variants were found segregating in many Hispanic and non‐Hispanic families.

## 1 BACKGROUND

In the United States (US) in 2023, Alzheimer's disease (AD) was present in 6.7 million people, representing 10.8% of the population over age 65 years, and is projected to grow to 13.8 million people, nearly 3.3% of the total US population, by 2060.^1^ The cause or causes of AD remain elusive, but there is strong evidence that it is a polygenic disorder. Highly penetrant pathogenic variants in AD – *APP*,^2^, ^3^ *PSEN1^4^*, and *PSEN2* ^5^ – explain less than 5% of early‐onset familial AD (fAD). The *APOE* ε4 allele is associated with the risk of developing late‐onset, sporadic AD^6^, ^7^ and has been found in most ethnic groups.

sLate‐onset sporadic AD is highly heritable (∼70%), implying that genetic factors contribute to its etiology. The largest genome‐wide association studies (GWASs) that included thousands of unrelated individuals with AD and non‐demented controls led by the European Alzheimer and Dementia Biobank Consortium, the UK Biobank, the Alzheimer's Disease Genetic Consortium, the Alzheimer's Disease Sequencing Project, FinnGen, and CHARGE Consortia identified common variants in more than 75 genetic risk loci. None of these risk loci reaches the effect size of *APOE* ε4.^8^

The goal of this investigation was to identify rare variants associated within the 75 genetic loci in late‐onset fAD. We chose to limit the age at onset to later than 65 years to distinguish the study population from those with early‐onset fAD. This approach would allow us to estimate the proportion of families where the diagnosis of AD could be “explained” by genes identified by common variants in GWAS loci. Family‐based statistical tests are largely underpowered to detect rare variants in novel genes with a limited number of family members and little or no evidence of vertical transmission. Thus, we used a simple segregation analysis restricted to genes that were previously associated with AD in GWAS.

There are clear advantages to family‐based studies. Family‐based analyses reduce the effects of population stratification, allow for easier detection of Mendelian inconsistencies and accurate haplotype phasing, and can detect parent‐of‐origin effects. Investigation of well‐characterized and carefully selected families can result in the identification of rare variants with significant roles in the pathogenesis of fAD.^9^

One of the questions most frequently posed by family members of patients with fAD concerns familial risk. Polygenic risk scores have recently been used to assess recurrence risk and penetrance in families.^10^ However, identification of pathogenic variants in fAD would advance mechanistic and therapeutic research efforts and facilitate genetic counseling. To identify and determine the frequency of potentially pathogenic variants in fAD, we used genome sequencing data from families recruited in the US and the Dominican Republic to identify damaging missense and loss‐of‐function (LoF) variants in candidate genes nominated from the largest GWAS analyses of clinically and pathologically diagnosed AD.^8^

## 2 METHODS

### 2.1 Inclusion criteria and ethical review

We required first‐degree family members, typically siblings, as the primary criterion.^11^ The criteria for study entry included two living affected (clinically demented or cognitively symptomatic) siblings, ≥60 years old, and a third living relative, similar in age, with or without dementia. We also included families with deceased affected siblings, as long as autopsy information or frozen brain tissue was available for affected members. Families in which participants were symptomatic but did not meet criteria for AD were still included with the commitment for follow‐up. Details of the recruitment for both cohorts were previously described.^11^, ^12^, ^13^ Recruitment protocols underwent ethical review at each site and were consistent across all sites in the National Institute on Aging (NIA) Alzheimer's Disease Family Based Study (AD‐FBS). In the Dominican Republic, there was a central review board for the Estudio Familiar de la Influencia Genética en Alzheimer (EFIGA) study following the review at Columbia University. Women and men were included. There were no exclusions based on ethnic group or race because this was a family‐based study that investigated family‐specific rare variants.

### 2.2 NIA AD‐FBS

Beginning in 2002, families were recruited from Alzheimer's Disease Centers across the United States. Inclusion criteria for this study required one member to have had a diagnosis of definite or probable AD^14^ with onset after age 60 and a sibling with definite, probable, or possible AD and a similar age at onset. Another relative of the affected sibling pair, ≥60 years old if unaffected or diagnosed with AD if affected, was required. Any relatives over age 50 years were also recruited, regardless of cognitive status, but individuals deemed unaffected were required to have had documented cognitive testing and clinical examination. Most sites used a standard neuropsychological battery, including Logical Memory (Immediate and Delayed), Selective Reminding Task, Orientation (Mini‐Mental State Examination [MMSE]), Digit Span Forward and Backward, Digit Ordering, and Category Fluency (animals and vegetables).^11^ All participants provided informed consent. Demographic information, previous clinical diagnoses, age at onset for patients with AD, method of diagnosis, and Clinical Dementia Rating scale of 15 were ascertained. The age at onset for participants was defined as the age at which the family first observed signs of impaired cognition. For unaffected family members, we used their age at the time of their latest examination without impairment. Participants with advanced disease or those living in a remote location that could not complete a detailed in‐person evaluation contributed a blood sample, and the site investigator conducted a detailed review of medical records or an assessment by telephone or videoconference to document the presence or absence of AD. We used the National Institute on Aging‐Alzheimer Association criteria^14^ for definite AD or pathological criteria for AD^16^, ^17^ when based on *post mortem* information alone. Individuals with other forms of dementia, mild cognitive impairment, or uncorroborated family reports of AD were considered unaffected. From a pool of 251 families with 1055 individuals, we restricted the selection to families with two or more affected family members by clinical or pathological diagnosis, which yielded 197 families with 926 individuals and whole genome sequencing (WGS) data. There were 545 (58.8%) individuals with fAD and 381 individuals without dementia. The mean age at onset was 75.2 (SD 8.04) years old, and they were White, non‐Hispanic. Those affected were significantly older than those unaffected (Table 1A). The majority of families were White and of non‐Hispanic ancestry. There were approximately 12 families of African American ancestry.

**RESEARCH IN CONTEXT**

1. **Systematic review**: The goal of this study was to identify rare coding variants in genes segregating in families multiply affected by Alzheimer's disease among two different ethnic groups: White, non‐Hispanic and Caribbean Hispanic. The authors focused their search on variants located in or near statistically significant loci identified in genome‐wide association studies (GWASs). After reviewing several GWASs in the literature, the authors selected the largest GWAS to date.
2. **Interpretation**: Rare coding variants in several genes from GWASs segregated completely in 17.5% of families, while APOE e4 was the only segregating variant in 22.6% of families. Interestingly, in 248 (60.3%) families, neither *APOE*‐ε4 nor any segregating coding variants were found.
3. **Future directions**: A compelling argument can be made that efforts to identify rare variants in familial Alzheimer's disease should be accelerated. Such findings can provide critical information to families for counseling and facilitate mechanistic and therapeutic research efforts.

**TABLE 1A and 1B:** Distribution of participating families, age at onset, and APOE ε4 frequency.

1A. NIA AD‐FBS	Total families	Total participants	Affected	Unaffected	Age affected	Age unaffected	*APOE* ε4 (%)
Total families	251	1055	594	461	75.19 ± 8.03	71.61 ± 9.72	0.38
Less than two affected	54	79	60	19	76.18 ± 6.17	81 ± 2.94	0.42
Greater than two affected	197	926	545	381	75.21 ± 8.04	70.56 ± 9.41	0.38

1B. EFIGA	Total families	Total participants	Affected	Unaffected	Age Affected	Age Unaffected	*APOE* ε4 (%)
Total families	271	1480	833	647	71.97 ± 9.85	66.54 ± 8.77	0.33
Less than two affected	57	435	145	290	80.25 ± 8.66	81.28 ± 6.76	0.27
Greater than two affected	214	1340	793	547	71.91 ± 9.88	66.11 ± 8.72	0.33

### 2.3 EFIGA cohort

We acquired data from a second set of families with late‐onset fAD from the EFIGA cohort to identify variants segregating among families of Caribbean Hispanic ancestry. Study design, adjudication, and clinical assessment of AD in this cohort were described previously^12^, ^18^ and similar to those used in the AD‐FBS. The neuropsychological battery for EFIGA included the Selective Reminding Test, Benton Recognition, Orientation (from MMSE), Abstract Reasoning (similarities and identities/oddities), Naming (Boston Naming Test), Verbal Fluency “CFL,” Category Fluency (animals), Repetition, Comprehension, Benton Matching, Attention, and Literacy.^12^, ^18^ All individuals provided informed consent. Participants were followed every 2 years and evaluated using the same neuropsychological battery, structured medical and neurological examination, assessment of depression,^19^ and the Clinical Dementia Rating scale,^15^ and the research clinical diagnosis of AD was determined. There were 271 families with 1481 individuals, but when the selection of families was restricted to those with two or more affected family members, 214 (78.9%) families remained with 1340 individuals with available genome sequencing. There were 793 (59.2%) individuals who met the criteria for clinical fAD and 547 individuals without dementia. The mean age of onset was 71.2 years (SD 9.9). Those affected were significantly older that those unaffected (Table 1B). All individuals included were of Caribbean Hispanic ancestry.

### 2.4 Whole genome sequencing

The genome sequencing at the New York Genome Center and at the Uniformed Services University of the Health Sciences using one microgram of DNA, an Illumina PCR‐free library protocol, and sequenced on the Illumina HiSeq platform. We assessed sex mismatches and unexpected familial relationships prior to analyses. We harmonized the WGS from the two sites in the EFIGA and AD‐FBS families and jointly called variants to create a uniform analysis set. Genomes were sequenced to a mean coverage of 30×. Sequence data analysis was performed using an automated analysis pipeline that matched the Centers for Common Disease Genomics (CCDG) and TOPMed recommended best practices.^20^ Briefly, sequencing reads were aligned to the human reference, hs38DH, using Burrows‐Wheeler Alignment ‐ Maximal Exact Matches (BWA‐MEM) version 0.7.15. Variant calling was performed using the Genome Analysis Toolkit (GATK) best practices. Variant filtration was performed using Variant Quality Score Recalibration (VQSR) at tranche 99.6%, which identifies annotation profiles of variants that could be called with high confidence and assigns a score Variant Quality Score Log‐Odds (VQSLOD) to each variant. Variants passing the VQSR threshold were further filtered out for sample missingness (> 2%), depth of coverage (DP) < 10 and genotype quality (GQ) > 20. We then annotated high‐quality variants using Annotate Variation (ANNOVAR). Specifically, variants were annotated for population‐level frequency using Genome Aggregation Database (gnomAD), in silico function using Variant Effect Predictor (VEP), and variant conservation using Combined Annotation Dependent Depletion (CADD) score.

### 2.5 Segregation analysis and identification of damaging missense and loss of function variants

The search was limited to variants in genetic loci identified in the largest GWASs, where individuals were clinically or pathologically examined.^8^ This analysis included cohorts such as UK Biobank, where AD diagnosis was ascertained by proxy or family history. We also searched genes involved in early onset fAD: *APP*, *PSEN1 and PSEN2*, and *AKAP9*. The total number of genes or loci queried was 81 (Table S1). Segregation of variants was defined by presence in all affected family members and in an unaffected family member if they were at least 5 years younger than the mean age of onset of AD in the family. Damaging coding variants were prioritized: loss of function (LoF) variants (stoploss, stopgain, frameshift, or splice variants) or damaging missense with a high CADD > 20. Only variants considered rare in the general population (less than 1% in gnomAD) were included. The high allelic frequency of *APOE* ε4 in the general population and in AD required examination of the co‐segregation within families with and without a segregation of damaging missense or LoF variants. As for linkage disequilibrium (LD) computation, we computed LD between the statistically significant common variant at each locus reported^8^ and segregating rare variants using PLINK. We report the *r* ^2^ between the variants as a measure of LD.

## 3 RESULTS

There were a combined 522 families with 2535 individuals sequenced in the two datasets. After families with less than two affected family members were excluded, 411 families with 2266 individuals remained in the two datasets. In the AD‐FBS, 926 individuals (58.9%) met the criteria for definite or probable fAD (19.6% vs 80.4%, respectively) and the remaining family members were unaffected. In EFIGA, there were 1340 individuals in this family group, of which 59.1% met the criteria for probable fAD, and the remaining individuals were considered unaffected. Other demographics were age at recruitment, age at onset, and sex (Tables 1A and 1B).

### 3.1 Presence of damaging missense and LoF variants

**FIGURE 1:**
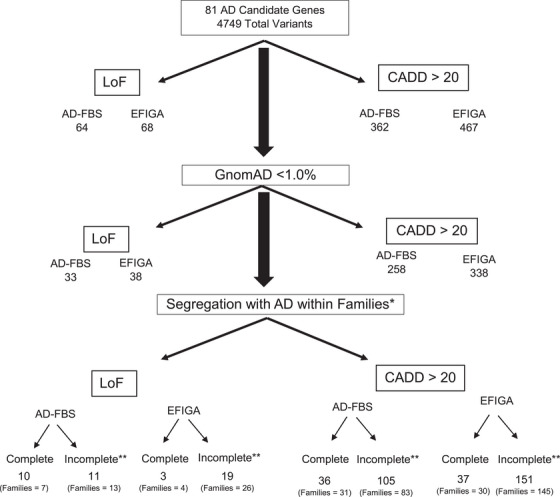
A filtering process was described in the methods to identify rare variants (gnomAD MAF less than 1%) with complete of incomplete segregation in families. Segregation was defined as being present in all affected members of a family. If a variant segregated in one family and showed incomplete segregation in another family, we required a qualifying variant to be seen only in affected members. Unaffected family members with qualifying variants were accepted if their age was ≥5 years below the age at onset of the affected carriers. **Incomplete segregation was defined as variants present in at least four affected individuals with AD in the specific cohort (AD‐FBS or EFIGA), observed in more affected than unaffected individuals, and if present in unaffected individuals if their age was ≥5 years below the age at onset of the affected carriers.

We identified 4749 total coding variants in 81 candidate genes and loci within the two cohorts (Table S1). We restricted the search to damaging potentially pathogenic coding variants using LoF variants or missense variants with a CADD score greater than 20 (Figure 1). Forty‐six damaging coding variants or LoF variants were identified in 32 genes among 38 White, non‐Hispanic families in AD‐FBS. In the EFIGA there were 40 coding variants in 32 genes segregating in 34 families. *ABCA1*, *AKAP9*, *ANK3*, *NYO15A*, *and SLC26A1* contained the highest number of variants. No damaging missense variant was present in both family datasets, but a rare LoF frameshift variant in *ABCA7* (Ch19:1044708, GGGGCACCTGGT:G) was present in the AD‐FBS and EFIGA datasets. A full list of the coding variants in both family datasets are presented in Table 2. Variants in 20 genes were also found in the case‐control series from the Alzheimer's Disease Sequencing Project (Table S2). Over 100 damaging missense or LoF variants in the AD‐FBS and in the EFIGA families showed incomplete segregation in families (Table S3).

**TABLE 2:** Distribution of damaging missense and loss‐of‐function variants by cohort and variant type.

Gene		Cohort	Variant type	Annotation
*ABCA1*	chr9:104804721:C:G chr9:104798567:T:C chr9:104817343:G:A chr9:104819624:C:T chr9:104884475:G:A	AD‐FBS EFIGA AD‐FBS AD‐FBS AD‐FBS	LoF Missense Missense Missense Missense	Member of superfamily of ATP‐binding cassette transporters
*ABCA7*	chr19:1057344:G:A chr19:1051965:C:T chr19:1044708:GGGGCACCTGGT:G	AD‐FBS EFIGA EFIGA/ AD‐FBS	Missense Missense LoF	Transmembrane transporter protein
*ABI3*	chr17:49217803:G:A	AD‐FBS	Missense	Member of an adaptor protein family, also associated with Lewy Body disease
*ACE*	chr17:63479054:G:C	EFIGA	Missense	Degradation and clearance of Aβ
*ADAM10*	chr15:58717601:C:T	EFIGA	Missense	Constitutive α‐secretase processing of APP^52^
*ADAMTS1*	chr21:26838049:G:A chr21:26838216:C:T	AD‐FBS EFIGA	Missense Missense	APP hydrolysis decreased Aβ
*ADAMTS20*	chr12:43356572:T:C chr12:43428519:A:G	EFIGA EFIGA	Missense Missense	A disintegrin‐like and metalloproteinase with thrombospondin type 1 motifs
*AKAP9*	chr7:91992989:G:C chr7:92097077:C:A chr7:92038422:A:G chr7:92040807:G:A chr7:92102769:G:A	AD‐FBS AD‐FBS EFIGA EFIGA EFIGA	Missense Missense Missense	Enhances tau phosphorylation
*ANK3*	chr10:60070700:T:A chr10:60073965:T:C chr10:60074069:A:C chr10:60075299:G:A chr10:60186803:G:A	EFIGA EFIGA AD‐FBS AD‐FBS AD‐FBS	Missense Missense Missense Missense Missense	Alters methylation dynamics associated with aging specifically in neurons
*BCKDK*	chr16:31110471:C:T	EFIGA	Missense	Associated with obesity and expression of AD related genes
*CR1*	chr1:207511589:C:T chr1:207523952:C:T	EFIGA EFIGA	Missense	Immune dysfunction and glial‐mediated neuroinflammation
*CTSB*	chr8:11847108:T:G	AD‐FBS	Missense	Accumulates in amyloid plaques in human AD brain
*DOC2A*	chr16:30010237:T:C	AD‐FBS	LoF	Microglial regulator of CNS innate immunity found in AD
*ECHDC3*	chr10:11742730:C:T	EFIGA	LoF	Enoyl‐CoA hydratase Domain containing 3 involved in lipid metabolism
*EED*	chr11:86245326:C:T chr11:86245267:C:T	EFIGA AD‐FBS	Missense Missense	EED variants impact LTP, a measure of hippocampal memory function
*EPHA1*	chr7:143398406:G:A chr7:143391696:G:A	AD‐FBS EFIGA	Missense Missense	Eph/ephrin signaling disrupts and alters activity at blood–brain barrier
*GPR141*	chr7:37741061:C:A chr7:37741003:A:T	EFIGA AD‐FBS	Missense Missense	Encodes an orphan receptor of Class A rhodopsin‐like G protein‐coupled receptors
*HS3ST5*	chr6:114057958:G:A	AD‐FBS	Missense	Enhances spread of tau pathology^61^
*IDUA*	chr4:987896:C:G	EFIGA/ AD‐FBS	Missense Missense	Accumulation of glycosaminoglycans
*IL34*	chr16:70654657:C:A	AD‐FBS	LoF	Physiological role in clearance of cerebral Aβ protein
*LILRB2*	chr19:54279530:T:A	EFIGA	Missense	LILRB2 antagonist antibody on microglial responses to amyloid plaques^64^
*MYO15A*	chr17:18120185:G:A chr17:18173824:G:A chr17:18119509:G:A chr17:18120434:C:T chr17:18133274:G:A chr17:18142122:G:A	EFIGA EFIGA AD‐FBS AD‐FBS AD‐FBS AD‐FBS	Missense Missense Missense Missense Missense Missense	Endosomal compartment dysfunction
*NME8*	chr7:37884404:G:T chr7:37896956:C:CA	EFIGA AD‐FBS	LoF	Protective and antioxidant action
*NYAP1*	chr7:100490675:C:T chr7:100488976:C:T	AD‐FBS EFIGA	Missense Missense	Affects PILRB and PILRA immunoglobulin‐like receptors
*PLCG2*	chr16:81858323:C:T chr16:81923501:A:G	EFIGA AD‐FBS	Missense Missense	Inflammatory response induced by amyloid plaques
*PRDM7*	chr16:90060590:C:G chr16:90062060:A:G	AD‐FBS	Missense	Transcription and other nuclear processes
*PRKD3*	chr2:37289451:T:TG chr2:37289452:TACTC:T chr2:37289460:T:TTG	AD‐FBS	LoF	Inflammatory signaling
*PSEN1*	chr14:73170945:C:T chr14:73170959:A:G	AD‐FBS EFIGA	Missense Missense	Subunit of γ‐secretase
*PTK2B*	chr8:27437198:G:A	EFIGA	Missense	Pyk2 suppresses tau phosphorylation
*RBCK1*	chr20:427337:C:T	AD‐FBS	LoF	Heterologous ubiquitination
*SFRP4*	chr7:37916317:C:T	EFIGA	Missense	Oxidative stress and inflammation
*SLC26A1*	chr4:991652:G:A chr4:989768:G:A chr4:989896:A:G chr4:989305:G:A chr4:990080:G:A chr4:990241:C:T	AD‐FBS EFIGA EFIGA AD‐FBS AD‐FBS AD‐FBS	LoF Missense Missense Missense Missense Missense	Member sulfate/anion transporter gene family
*SNX1*	chr15:64126203:C:T chr15:64131812:C:T	AD‐FBS AD‐FBS	Missense Missense	Member of retromer complex
*SORL1*	chr11:121604205:C:T	EFIGA	Missense	Endosomal degradation and recycling in neurons
*SPPL2A*	chr15:50722170:A:T	AD‐FBS	Missense	Involved in intramembrane cleavage of TMEM106B
*TNIP1*	chr5:151052238:T:C	AD‐FBS	Missense	Autoimmunity and tissue homeostasis via regulation of NFκB activation
*TPCN1*	chr12:113293011:C:T chr12:113288795:G:A	EFIGA AD‐FBS	Missense Missense	Lysosomal TPCN inhibition ameliorates Aβ pathology
*TREML2*	chr6:41198273:C:A	EFIGA	Missense	Hyperphosphorylation and aggregation of tau and induces activation of microglia
*TSPOAP1*	chr17:58307871:C:G chr17:58325608:C:T chr17:58327896:G:A	EFIGA EFIGA EFIGA	Missense	Phosphatidylglycerol‐regulated functions
*UNC5CL*	chr6:41033992:C:T	EFIGA	Missense	Differential methylation related to Aβ plaques
*USP6NL*	chr10:11463631:G:A	AD‐FBS	Missense	Acts as GTPase‐activating protein for Ras‐related protein
*WDR81*	chr17:1732823:G:A chr17:1728303:G:C	EFIGA AD‐FBS	Missense Missense	Promotes autophagic clearance of protein aggregates

Abbreviations: AD‐FBS, Alzheimer's disease – National Institute on Aging Family Based Study; EFIGA, Estudio Familiar de la Genética de la Enfermedad; LoF, loss of function.

### 3.2 *APOE* co‐segregation

*APOE* ε4 co‐segregated with LoF variants in *ABCA1*, *DOC2A*, and SLC26A1/IDUA in four of the White, non‐Hispanic families, but there was no co‐segregation in Hispanic families with any LoF variant. Missense variants in nine genes co‐segregated with *APOE* ε4, including *ABCA1*, *AKAP9*, *ANK3*, *CTSB*, *EED*, *PRDM7*, *SNX1*, *USP6NL*, and *WDR81* in the AD‐FBS families, while in the EFIGA families *APOE* ε4 segregated with missense variants in *ADAMTS20*, *AKAP9*, *ANK3*, *CR1*, *SORL1*, and *TSPOAP1*. The presence of *APOE* ε4 did not affect age at onset of dementia (Table 3). *APOE* ε4 was the only variant segregating 93 (22.6%) of families, Among the AD‐FBS cohort, 64 (32.5%) families had only segregation with *APOE* ε4, and in the EFIGA cohort, 29 (13.5%) families had only *APOE* ε4 segregation.

**TABLE 3:** Details of variants that completely segregate in at least one family.

Variant type	Cohort	SNP	Gene	Family variant completely segregates	AD patients in families where variant completely segregates	Healthy members in families where variant completely segregates*	Co‐segregates with APOE	AD GWAS index SNP	Family members that also carry GWAS index SNP
Frameshift variant	EFIGA	chr19:1044708:GGGG CACCTGGT:G	*ABCA7*	2	8	2	No	chr19:1050875:A:G	2
Stop gain		chr10:11742730:C:T	*ECHDC3*	1	3	0	No	chr10:11676714:A:G	3
Stop gain		chr7:37884404:G:T	*NME8*	1	2	1	No	chr7:37844191:T:C	0
Frameshift variant	AD‐FBS	chr19:1044708:GGGG CACCTGGT:G	*ABCA7*	1	3	1	No	chr19:1050875:A:G	0
Frameshift variant		chr7:37896956:C:CA	*NME8*	1	3	0	No	chr7:37844191:T:C	0
Splice acceptor variant		chr16:30010237:T:C	*DOC2A*	1	2	0	Yes	chr16:30010081:C:T	0
Structural interaction variant		chr16:70654657:C:A	*IL34*	2	5	2	Yes	chr16:70660097:C:A	0
Frameshift variant		chr2:37289451:T:TG	*PRKD3*	1	2	1	No	chr2:37304796:T:C	0
Frameshift variant		chr2:37289452:TACTC:T	*PRKD3*	1	2	1	No	chr2:37304796:T:C	0
Frameshift variant		chr2:37289460:T:TTG	*PRKD3*	1	2	1	No	chr2:37304796:T:C	0
Stop gain		chr20:427337:C:T	*RBCK1*	1	2	0	No	chr20:413334:A:G	2
Stop gain		chr4:991652:G:A	*SLC26A1/IDUA*	1	2	0	Yes		0
Splice acceptor variant		chr9:104804721:C:G	*ABCA1*	1	2	0	Yes	chr9:104903697:C:G	0
Damaging missense (CADD > 20)	EFIGA	chr19:1051965:C:T	*ABCA7*	1	2	4	No	chr19:1050875:A:G	1
		chr17:63479054:G:C	*ACE*	1	2	0	No	chr17:63471557:C:T	2
		chr15:58717601:C:T	*ADAM10*	1	2	2	No	chr15:58764824:T:A	4
		chr21:26838216:C:T	*ADAMTS1*	2	4	0	No	chr21:26775872:C:T	4
		chr12:43356572:T:C	*ADAMTS20*	1	5	3	No	chr12:43573874:T:G	0
		chr12:43428519:A:G	*ADAMTS20*	1	2	1	Yes	chr12:43573874:T:G	3
		chr7:92038422:A:G	*AKAP9*	1	2	0	No		
		chr7:92040807:G:A	*AKAP9*	1	2	0	No		
		chr7:92102769:G:A	*AKAP9*	1	2	0	No		
		chr1:207511589:C:T	*CR1*	1	2	1	Yes	chr1:207577223:T:C	3
		chr1:207523952:C:T	*CR1*	1	5	6	No	chr1:207577223:T:C	2
Damaging missense (CADD > 20)	EFIGA	chr7:143391696:G:A	*EPHA1*	1	2	3	No	chr7:143413669:G:A	3
		chr7:100488976:C:T	*NYAP1*	1	2	2	No	chr7:100494172:T:C	4
		chr14:73170959:A:G	*PSEN1*	1	5	1	No		
		chr8:27437198:G:A	*PTK2B*	1	2	0	No	chr8:27362470:C:T	0
		chr11:121604205:C:T	*SORL1*	1	3	0	Yes	chr11:121564878:T:C	0
		chr4:987896:C:G	*IDUA*	1	2	0	No	chr4:993555:G:T	0
		chr4:989768:G:A	*SLC26A1/IDUA*	1	2	0	No	chr4:993555:G:T	0
		chr4:989896:A:G	*SLC26A1/IDUA*	1	3	0	No	chr4:993555:G:T	0
		chr6:41033992:C:T	*UNC5CL*	1	3	0	No	chr6:41036354:G:A	0
		chr6:41198273:C:A	*TREML2*	1	4	1	No	chr6:41181270:A:G	0
		chr7:37741061:C:A	*GPR141/EPDR1*	1	2	0	Yes	chr7:37844191:T:C	2
		chr7:37916317:C:T	*SFRP4/EPDR1*	1	2	0	No	chr7:37844191:T:C	2
		chr9:104798567:T:C	*ABCA1*	1	3	0	Yes	chr9:104903697:C:G	3
		chr10:60070700:T:A	*ANK3*	1	2	1	Yes	chr10:60025170:T:G	2
		chr10:60073965:T:C	*ANK3*	1	3	0	No	chr10:60025170:T:G	0
		chr11:86245326:C:T	*EED*	1	3	0	No	chr11:86157598:T:C	3
		chr12:113293011:C:T	*TPCN1*	1	3	0	No	chr12:113281983:T:C	3
		chr16:31110471:C:T	*BCKDK*	1	2	0	No	chr16:31111250:C:T	0
		chr16:81858323:C:T	*PLCG2*	1	2	0	No	chr16:81908423:C:G	0
		chr17:1732823:G:A	*WDR81*	1	4	0	No	chr17:1728046:TGAG:T	0
		chr17:18120185:G:A	*MYO15A*	1	3	0	No	chr17:18156140:G:A	0
		chr17:18173824:G:A	*MYO15A*	1	2	0	No	chr17:18156140:G:A	0
		chr17:58307871:C:G	*TSPOAP1*	1	4	1	No	chr17:58332680:A:G	3
		chr17:58325608:C:T	*TSPOAP1*	1	4	0	Yes	chr17:58332680:A:G	0
		chr17:58327896:G:A	*TSPOAP1*	1	2	2	No	chr17:58332680:A:G	2
		chr19:54279530:T:A	*LILRB2*	1	2	0	No	chr19:54267597:C:T	1
Damaging missense (CADD > 20)	AD‐FBS	chr19:1057344:G:A	*ABCA7*	2	5	4	No	chr19:1050875:A:G	6
		chr21:26838049:G:A	*ADAMTS1*	1	4	2	No	chr21:26775872:C:T	6
		chr7:91992989:G:C	*AKAP9*	1	2	0	Yes		
		chr7:92097077:C:A	*AKAP9*	1	3	2	No		
		chr7:143398406:G:A	*EPHA1*	1	3	1	No	chr7:143402040:C:A	2
		chr7:100490675:C:T	*NYAP1*	1	2	6	No	chr7:100494172:T:C	1
		chr14:73170945:C:T	*PSEN1*	1	2	0	No		
		chr15:50722170:A:T	*SPPL2A*	1	4	0	No	chr15:50716490:T:C	4
		chr4:987896:C:G	*IDUA*	1	2	0	No	chr4:993555:G:T	0
		chr4:989305:G:A	*SLC26A1/IDUA*	1	2	1	No	chr4:993555:G:T	0
		chr4:990080:G:A	*SLC26A1/IDUA*	1	2	0	No	chr4:993555:G:T	0
		chr4:990241:C:T	*SLC26A1/IDUA*	1	2	0	No	chr4:993555:G:T	0
		chr7:37741003:A:T	*GPR141/EPDR1*	1	3	1	No	chr7:37844191:T:C	0
		chr9:104817343:G:A	*ABCA1*	1	3	1	No	chr9:104903697:C:G	4
		chr9:104819624:C:T	*ABCA1*	1	2	0	Yes	chr9:104903697:C:G	0
		chr9:104884475:G:A	*ABCA1*	1	2	0	No	chr9:104903697:C:G	0
		chr10:60074069:A:C	*ANK3*	1	3	0	Yes	chr10:60025170:T:G	3
		chr10:60075299:G:A	*ANK3*	1	2	1	Yes	chr10:60025170:T:G	1
		chr10:60186803:G:A	*ANK3*	1	2	0	Yes	chr10:60025170:T:G	2
		chr11:86245267:C:T	*EED*	1	3	4	Yes	chr11:86157598:T:C	3
		chr12:113288795:G:A	*TPCN1*	1	2	0	Yes	chr12:113281983:T:C	0
		chr16:81923501:A:G	*PLCG2*	1	2	0	No	chr16:81908423:C:G	0
		chr17:1728303:G:C	*WDR81*	1	2	0	Yes	chr17:1728046:TGAG:T	0
		chr17:18119509:G:A	*MYO15A*	1	2	0	No	chr17:18156140:G:A	2
		chr17:18120434:C:T	*MYO15A*	1	2	0	No	chr17:18156140:G:A	0
		chr17:18133274:G:A	*MYO15A*	1	2	1	No	chr17:18156140:G:A	0
		chr17:18142122:G:A	*MYO15A*	1	2	0	No	chr17:18156140:G:A	0
		chr10:11463631:G:A	*USP6NL*	1	2	0	Yes	chr10:11676714:A:G	0
		chr15:64126203:C:T	*SNX1*	1	3	1	No	chr15:64131307:G:A	1
		chr15:64131812:C:T	*SNX1*	1	2	0	Yes	chr15:64131307:G:A	0
		chr16:90060590:C:G	*PRDM7*	1	2	0	Yes	chr16:90103687:G:A	0
		chr16:90062060:A:G	*PRDM7*	1	3	0	Yes	chr16:90103687:G:A	3
		chr17:49217803:G:A	*ABI3*	1	2	0	Yes	chr17:49219935:T:C	0
		chr5:151052238:T:C	*TNIP1*	1	2	0	No	chr5:151052827:C:T	0
		chr6:114057958:G:A	*HS3ST5*	1	2	1	No	chr6:114291731:T:C	0
		chr8:11847108:T:G	*CTSB*	1	3	4	Yes	chr8:11844613:G:C	0

### 3.3 Linkage disequilibrium (LD) between common and rare variants

Thirty‐three (43.4%) of the 76 damaging missense variants or LoF variants were in linkage disequilibrium with the most significant common variant from the GWAS^8^ or with any of the four other candidate genes. Because LD is sensitive to allele frequency, we anticipated that the likelihood of a common and rare variant being in LD would be very low. Thus, we also investigated whether the most significant common variant from the GWAS data co‐segregated with any rare variant we identified. Within AD‐FBS families, we identified one LoF variant in *RBCK1* and ten damaging missense variants that co‐segregated with the common variant (Table 3). Within EFIGA families, 15 damaging missense variants and one LoF variant completely co‐segregated with the common variants (Table 3).

In 64 (32.5%) of the AD‐FBS families and 29 (13.6%) of the EFIGA families, there were no segregating variants in any of the GWAS loci and *APOE* ε4 was the only risk allele segregating in these families. Among the AD‐FBS cohort, 99 (50.3%) families had no rare variants identified and were not found to have *APOE* ε4 accounting for the segregation within the families. Similarly, 149(69.6%) of the EFIGA families had no rare variants identified and did not have *APOE* ε4 segregation.

## 4 DISCUSSION

Using existing data from whole genome sequencing in two groups of families multiply affected by late onset fAD we identified rare, potentially pathogenic, segregating variants embedded in or near risk loci reported from a large GWAS of unrelated individuals^8^, present in Mendelian forms of early onset fAD,^2^, ^3^, ^4^, ^5^ or identified by small sequencing investigations (e.g., *AKAP9)*.^21^ We found numerous coding variants prior to restricting the analyses to damaging missense variants and LoF variants at these loci. In 38 AD‐FBS families, variants in 29 genes were identified as fully segregating in all affected family members and younger relatives at risk (Table 3). Similarly, in 34 EFIGA families, variants in 31 genes were observed segregating. The highest frequency of segregating variants was found in *ABCA1*, *AKAP9*, *ANK3*, *MYO15A*, *and SLC26A1* in both groups of families. We also found no segregating variants within the GWAS loci and no evidence of *APOE* ε4 segregation in 60.3% of the families. This clearly indicates that an unbiased search for additional pathogenic variants, including structural variants, should be undertaken to fully explain the genetic basis of fAD.

Genome‐wide array studies were designed using common genetic variation across the genome to identify loci that are associated with a trait or disease. Most are common variants that confer small effects regarding the proportion of the trait or disease risk. Rare variants are defined by an allele frequency of less than 0.01. This is an arbitrary criterion, but it is widely accepted in the field and based on the observed effect sizes for variants at different minor allele frequency (MAF) thresholds.^22^ Rare variants generally have a larger effect size compared with common variants, and they are more suitable for functional genomic and mechanistic analyses investigating them as potential drug targets. They can also be used for genetic counseling or as putative biomarkers to support a personalized medical treatment.^23^

Determining whether the rare variants identified in these two cohorts were in LD with the common variants from the GWAS loci can be difficult because the much higher frequency of the significant common variant compared with the rare variant. As an alternative we determined the frequency by which the significant common variant in the GWAS co‐segregated with the rare variant. Among the AD‐FBS families, one (10%) LoF variant and 10 (41.7%) missense variants co‐segregated with the significant common variant, compared with two (66.7%) LoF variants and 16 (57.1%) missense variants that co‐segregated with the significant common variant in EFIGA families (Table 2).

Rare variants were found in *ABCA1*, *AKAP9*, and *ABCA7*, which are well established as genes involved in AD. A variant in *ABCA1*, an adenosine triphospate (ATP)‐binding cassette transporter A1, was identified in a large exome sequencing study^24^ of AD and associated with reduced ApoE levels and decreased clearance of Aβ.^25^ This specific variant was not observed in the family data, but several rare variants in *ABCA1* co‐segregated with chr9: 104794495 (Table 3). Rare variants in A‐kinase anchoring protein 9, *AKAP9*, were described in a small cohort of black, non‐Hispanic individuals.^21^ Subsequently, a rare variant (p.Arg434Trp) was observed in two large families from the Dominican Republic.^13^ *AKAP9* contains 51 exons and is a binding protein that connects protein kinase to intracellular locations promoting cAMP‐related activation of PKA, which in turn enhances tau phosphorylation.^26^ This process is one of the major findings in *post mortem* AD brains. Although only the ATP‐binding cassette, subfamily A, member 7 (ABCA7) gene was first identified using common variants in a GWAS27, it has both common risk variants with functional consequences and rare, damaging coding variants. *ABCA7* is a large gene with 47 exons encoding a transmembrane transporter protein. *ABCA7* is expressed in the brain, especially in microglia, neurons, endothelial cells, and pericytes, where it may help degrade Aβ.^28^ It might also play an important role in lipid metabolism. Common variants in *ABCA7* associated with AD have been observed in Black and White, non‐Hispanic individuals as well as in Hispanics.^27^, ^28^ There have also been family data where premature termination codons were found in *ABCA7*.^29^

The *ANK3* gene encodes for multiple isoforms of the scaffold protein Ankyrin G, which is involved in protein interactions and neuronal functions. Mutations in ANK3 alter neuronal excitability and connectivity by altering ion channels and membrane proteins.^30^ *ANK1* has been found to be differentially methylated in AD. Several variants were identified in MYO15A, which encodes a myosin involved in actin organization in mechanosensory hair bundles. Actin is involved in the retromer function, which occurs in several neurodegenerative diseases including AD. SLC26A1 encodes a protein that acts as a transporter for ions and organic substrates.^32^ Variants in SLC26A1 may disrupt the function of the gene leading to oxidative stress, inflammation, and metabolic dysfunction.^33^

Rare variants provide hypothesis‐free evidence for causality and can be targets for functional analyses to understand disease mechanisms.^23^ Thus, while variants in several other AD genetic loci were less frequent, they are no less important. Several lines of evidence have implicated the angiotensin converting enzyme, *ACE*, in AD.^34^, ^35^ *ACE* encodes ACE‐1, a rate‐limiting enzyme in the cRAS pathway that generates disease‐associated angiotensin II (Ang II) but also degrades and facilitates the clearance of Aβ. The gene encoding disintegrin‐like and metalloproteinase with thrombospondin type 1 (*ADAMTS1*) is a member of the ADAMTS protein family. This family of genes is distinguished from *ADAM10* based on the number of copies of the thrombospondin 1‐like repeats. Both are involved in extracellular matrix damage and repair.^36^ *ADAMTS1* is thought to be involved in the hydrolysis of APP and consequently decreases Aβ generation by inhibiting β‐secretase‐mediated cleavage. Clinically the gene may also affect cognitive ability by its effect on hippocampal amyloid processing. *CR1*, which encodes a complement receptor, regulates the complement cascade to clear cellular debris, including Aβ.^37^ ECHDC3 encodes an enoyl CoA hydratase domain containing enzyme, and is a gene known to be involved in plasma lipids and other lipid‐related traits. Its expression is altered in the brains of patients with AD.^38^ *EPHA1* belongs to the ephrin receptor subfamily of the protein‐kinase family, and these receptors have been involved in the development of the nervous system. In an early GWAS of AD, the Alzheimer's Disease Genetics Consortium identified a common variant in *EPHA1* associated with AD. Gene Ontology annotations related to this gene include transferase activity, transferring phosphorus‐containing groups, and protein tyrosine kinase activity.^39^ Variants in neuronal tyrosine phosphorylated phosphoinositide‐3‐kinase adaptor 1, *NYAP1*, appear to regulate immunoglobulin‐like receptors beta and alpha (PILRB and PILRA), ^40^ but it is unclear how these variants contribute to AD pathogenesis. Presenilin 1, *PSEN1*, a gene associated with early onset AD, was previously observed in late‐onset fAD,^41^, ^42^ and it encodes the catalytic subunit of α‐secretase involved in Aβ processing.

Variants in protein tyrosine kinase 2 beta, *PTK2B*, are thought to disrupt the normal suppression of tau phosphorylation and the effects of tauopathy, thereby adding to the pathogenesis of AD.^43^, ^44^, ^45^ Sortilin‐related receptor with A‐type repeats (SORLA or SORL1) is a sorting receptor in which variants impair processing of APP to soluble (s)APP and to the amyloid beta‐peptide. SORL1 is also involved in the endosomal degradation of Aβ and recycling in neurons.^24^, ^46^ Finally, a common variant in signal peptide peptidase‐like 2A, *SPPL2A*, was identified in a large genome‐wide array, and the gene is thought to be part of the immune response.^8^, ^47^ We annotated the function of other genes with segregating variants in Table 2.

Rare variants have distinctive features and a low likelihood of LD with other variants in the region, are more likely to affect gene function or expression, and play a more direct role in diseases.^23^ Efforts to discover associations driven by low‐frequency, rare variants through genome sequencing efforts provide refinements to estimates of disease heritability.^48^ Identifying rare variants in protein‐coding regions through genome sequencing would also greatly facilitate annotating genes associated with complex disease and describing the functional consequences of human sequence variation.

The reduction in costs for genetic testing will also make it more feasible to include in genome or exome sequencing in clinical practice. Strategies to identify damaging variants will soon be in place, and clinicians and counselors will need to be able to identify and explain these results to patients and families. Families generally want to know the diagnostic relevance of our findings and the potential recurrence risk to other family. Therefore, a compelling argument can be made to accelerate efforts to identify rare variants in fAD because of the relative importance of turning these discoveries into meaningful clinical and biological progress.

## CONFLICT OF INTEREST STATEMENT

All of the authors receive funding from the National Institutes of Health. The following authors have no other relevant conflicts of interest to report: Tamil Iniyan Gunasekaran, Dolly Reyes‐Dumeyer, Kelley M. Faber, Debby Tsuang, Diones Rivera Mejia, Martin Medrano, Rafael A. Lantigua, Margaret Pericak‐Vance, Jonathan L. Haines, Robert Sweet, Carlos Cruchaga, Camille Alba, Clifton Dalgard, Tatiana Foroud, and Richard Mayeux.

Alison Goate receives money from Athena Diagnostics for licensing of TDP43 mutations and has consulted for UK Dementia Research Institute, UK VIB, Katholik University, Leuven, Belgium Center for Molecular Neurology, Antwerp, Belgium Queensland Brain Institute, Brisbane, Australia.

Brad Boeve receives honoraria for SAB activities for the Tau Consortium, which is funded by the Rainwater Charitable Foundation; research support for clinical trials sponsored by Alector, Biogen, Transposon, Cognition Therapeutics, and EIP Pharma; and research support from the Lewy Body Dementia Association, American Brain Foundation, Dorothy and Harry T. Mangurian Jr. Lewy Body Dementia Program, the Little Family Foundation, and the Turner Family Foundation.

Roger Rosenberg receives funds from the Zale Foundation and license/royalty fees from Elsevier Publishing Inc., Springer Publishing Inc.; payments from Elsevier, Springer and Vitruvian, Inc., and The American Academy of Neurology. In addition, he has a 2009 patent on an Amyloid Beta Gene vaccine.

Debby Tsuang receives consulting fees from Acadia Pharma.

David Bennett has consulting relationships with AbbVie Inc.

Author disclosures are available in the supporting information.

## CONSENT STATEMENT

NIA AD‐FBS. All institutions were required to have their local Institutional Review Board approve the consent form for this investigation. Thus, all individuals provided informed consent.

EFIGA. The Dominican Republic has a centralized Institutional Review Board. They approved the consent form used in this investigation for families living in the Dominican Republic. Thus, all individuals provided informed consent.

## Supporting information

Supporting Information

Supporting Information
